# Calcium Transport and Enrichment in Microorganisms: A Review

**DOI:** 10.3390/foods13223612

**Published:** 2024-11-12

**Authors:** Hai Zhou, Yan-Yu Hu, Zhen-Xing Tang, Zhong-Bao Jiang, Jie Huang, Tian Zhang, Hui-Yang Shen, Xin-Pei Ye, Xuan-Ya Huang, Xiang Wang, Ting Zhou, Xue-Lian Bai, Qin Zhu, Lu-E Shi

**Affiliations:** 1 Department of Biotechnology, College of Life and Environmental Sciences, Hangzhou Normal University, Hangzhou 311121, China; 2021210315063@stu.hznu.edu.cn (H.Z.); huyanyu@stu.hznu.edu.cn (Y.-Y.H.); 2024112010025@stu.hznu.edu.cn (Z.-B.J.); 2021210315061@stu.hznu.edu.cn (J.H.); 2021210315296@stu.hznu.edu.cn (T.Z.); 2024112010011@stu.hznu.edu.cn (H.-Y.S.); 2023111010050@stu.hznu.edu.cn (X.-P.Y.); 2022111010066@stu.hznu.edu.cn (X.-Y.H.); 2022210301044@stu.hznu.edu.cn (X.W.); zt20100061@163.com (T.Z.); baixl2012@163.com (X.-L.B.); qinz1981@gmail.com (Q.Z.); 2 School of Culinary Art, Tourism College of Zhejiang, Hangzhou 311231, China

**Keywords:** calcium, enrichment, mechanism, transport, application

## Abstract

Calcium is a vital trace element for the human body, and its deficiency can result in a range of pathological conditions, including rickets and osteoporosis. Despite the numerous types of calcium supplements currently available on the market, these products are afflicted with a number of inherent deficiencies, such as low calcium content, poor aqueous solubility, and low human absorption rate. Many microorganisms, particularly beneficial microorganisms, including edible fungi, lactic acid bacteria, and yeast, are capable of absorbing and enriching calcium, a phenomenon that has been widely documented. This opens the door to the potential utilization of microorganisms as novel calcium enrichment carriers. However, the investigation of calcium-rich foods from microorganisms still faces many obstacles, including a poor understanding of calcium metabolic pathways in microorganisms, a relatively low calcium enrichment rate, and the slow growth of strains. Therefore, in order to promote the development of calcium-rich products from microorganisms, this paper provides an overview of the impacts of calcium addition on strain growth, calcium enrichment rate, antioxidant system, and secondary metabolite production. Additionally, it highlights calcium transport and enrichment mechanisms in microorganism cells and offers a detailed account of the progress made on calcium-binding proteins, calcium transport pathways, and calcium storage and release. This paper offers insights for further research on the relevant calcium enrichment in microorganism cells.

## 1. Introduction

Calcium is one of the essential vital elements for the human body, playing a crucial role in maintaining bone and dental health. The majority of calcium, approximately 99%, is predominantly located within the skeletal structure, while the remaining 1.0% is distributed throughout the blood, tissues, and organs, maintaining a dynamic equilibrium [1,2]. Calcium is also involved in a number of physiological processes, such as cell differentiation, enzyme activation, immune response, muscle contraction, synaptic transmission, hormone metabolism, and bone development [3,4]. Ingestion of excessive quantities of Ca^2+^ can result in the formation of urinary stones. Conversely, inadequate consumption may lead to the occurrence of a number of diseases, including rickets, muscle cramps, and hypertension [5,6,7]. The results of both animal and human studies have demonstrated that a moderate calcium intake can effectively mitigate postpartum hypertension, lower blood pressure, reduce the risk of dental caries, and serve as a preventive measure against osteoporosis and colon cancer [8,9].

Calcium deficiency has become a common society phenomenon, which can result from insufficient daily intake, reduced calcium bioavailability, and elevated calcium requirements within the human body [10]. Over the past decade years in Canada, there has been a significant decline in calcium intake, primarily due to reduced consumption of dairy products and concerns about the potential cardiovascular risks associated with calcium supplementation [11]. Similarly, in China, between 2019 and 2021, children aged 2–5 years exhibited significant deficits in dietary calcium intake, particularly in rural regions [12]. Furthermore, the research conducted among adults aged 18–35 across 15 provinces, municipalities directly under the central government of China, and autonomous regions revealed a declining trend in the dietary intake of nutrients, including calcium, phosphorus, sodium, potassium, iron, magnesium, copper, zinc, and manganese from 1989 to 2018 [13]. Osteoporosis is a condition that is defined by an increased susceptibility to fractures. This is attributed to a number of factors, including diminished bone mineral density, compromised bone quality, altered bone microstructure, and heightened bone fragility [14]. China has the highest number of individuals with osteoporosis worldwide, representing nearly half of the global total. Statistical data indicate that the prevalence of osteoporosis in China stands at 12.4%, with over 170 million individuals affected. The “Healthy China 2030” strategic framework explicitly outlines the implementation of a specialized initiative focusing on promoting bone health. Therefore, the consumer demand for calcium supplements has increased significantly, and the market for calcium supplementation health products continues to expand [15,16].

It is well established that supplemental calcium intake plays a pivotal role in the prevention and management of conditions associated with calcium deficiency, such as osteoporosis [5]. Various calcium formulations are available on the market. The initial calcium supplements included calcium lactate and calcium gluconate, which are characterized by low calcium content and low calcium absorption rates. New calcium supplements, such as ultrafine powdered calcium carbonate, peptide calcium chelates, and calcium amino acid preparations, have been developed. These supplements are characterized by enhanced solubility, good absorption rates, excellent bioavailability, and minimal gastrointestinal side effects [4,10,17]. Despite a number of calcium preparations currently available, they exhibit certain limitations, including low calcium content, poor water solubility, and limited absorption by the human body. Consequently, it is very necessary for carrying out a novel type of calcium agent with elevated calcium content, enhanced water solubility, and improved bioavailability [18,19]. Recent studies have demonstrated the remarkable potential of microorganisms, particularly those with beneficial properties, such as edible fungi, probiotics, yeast, and microalgae, to accumulate trace minerals [4,19,20,21]. The fortification of microorganisms with calcium ions represents a highly promising avenue of research, with the potential to develop a novel dietary supplement for calcium intake [19,22]. In consideration of the increasing demand for natural dietary supplements, calcium-enriched microorganisms represent a product with significant commercial potential. The most prevalent calcium accumulation method utilizes the addition of exogenous calcium salts into the substrate or fermentation medium. Consequently, calcium-enriched microorganisms have the potential to serve as a safe and effective source of daily calcium supplementation [19,23,24,25,26]. To the best of our knowledge, there is no review paper that focuses on calcium transport and enrichment in microorganisms. In order to develop microbial foods with high calcium levels, this paper conducted a review of the existing literature on calcium transport and enrichment in microbial cells. It focused on the impacts of calcium addition on strain growth, calcium enrichment rate, antioxidant system, and secondary metabolite production. Furthermore, the review elucidated the mechanisms of calcium transport and accumulation in microbial cells. It also provided a comprehensive overview of the advancements made in the fields of calcium-binding proteins, calcium transport pathways, and calcium storage and release mechanisms. This paper will provide a foundation for future research on calcium transport and accumulation within microorganism cells, with a particular focus on beneficial microorganisms.

## 2. Effects of Calcium Accumulation on Microbial Physiology

Calcium ions (Ca^2+^) function as second messengers in cells, enabling microorganisms to adapt to diverse environmental pressures through rapid perception and response to external stimuli [24]. Yang et al. [27] reported that the fungal calcium homeostasis system affected a number of essential life processes, including cell growth, conidial production, stress response, and the maintenance of normal cellular organelle function. However, excessively high concentrations of Ca^2+^ typically inhibit microbial growth [3,19,28]. Following the accumulation of Ca^2+^, significant changes are observed in microbial cell growth, metabolic compounds, and biological activities.

### 2.1. The Impact of Calcium Accumulation on Microbial Growth

Ca^2+^ is a critical macronutrient for living organisms, playing a pivotal role in microbial growth. In the microbiological industry, the microbial growth rate has a significant impact on process costs and efficiency [29]. Consequently, the research on the effects of Ca^2+^ accumulation on microbial growth is of paramount importance. Previous investigations have demonstrated that an optimal content of Ca^2+^ can facilitate the growth of mycelia and enhance the cultivation value of microorganisms, particularly edible fungi [30,31,32,33,34]. Hua et al. [31] found that the Ca^2+^ addition could accelerate the cell cycle of yeast *Candida tropicalis*, and the optimum concentration of Ca^2+^ was in the range of 10^−4^ to 10^−2^ mol/L. In a study by He et al. [3], the authors investigated the impacts of various calcium-containing compounds on the growth of *Pleurotus ostreatus* mycelia across a range of concentration gradients. Their findings revealed that the fungus exhibited optimal absorption capacity for calcium lactate at a concentration of 0.60 mg/mL, accompanied by the most rapid mycelial growth rate. Recently, He et al. [5] examined the capacity to enrich calcium through solid media and analyzed the impact of inorganic calcium supplementation on the growth of four edible fungi. The findings revealed that the addition of 200 mg/L of inorganic calcium to solid media significantly enhanced the proliferation of sulfur-bearing fungi. At an inorganic calcium concentration of 400 mg/L, the growth of *Poria cocos*, *Armillaria mellea*, and *Monascus purpureus* was promoted. However, an inorganic calcium content exceeding 600 mg/L had a negative impact on the growth of these four edible fungi. The research on calcium-rich mycelia revealed that at lower Ca^2+^ concentrations, the mycelia aggregated into spherical shapes [35,36,37], whereas Wu et al. [38] found that the addition of Ca^2+^ to the culture medium during the seed growth stage of *Penicillium griseofulvum* could stimulate the growth of spherical cells, with an increase in cell diameter as Ca^2+^ content rose. However, Higashiyama et al. [39] reported that spherical mycelia could impede oxygen absorption by the seed, thereby hindering mycelial growth. Conversely, Zhang et al. [37] postulated that a minor supplementation of Ca^2+^ induced the formation of spheres, enhancing oxygen mass transfer and facilitating mycelial growth. The formation of spherical mycelia might be associated with the quantity of Ca^2+^ introduced. The cell membrane plays a pivotal role in microbial growth, metabolism, and other biological processes. Ca^2+^ addition during the accumulation process may significantly affect microbial growth through the alteration of cell membranes. Momenijavid et al. [40] found that Ca^2+^ and alkaline pH could enhance the formation of *Escherichia coli* biofilms through a synergistic mechanism, namely that Ca(OH)_2_ could promote biofilm growth. Some investigations have demonstrated that the expression of the essential gene ALS3 for biofilm formation is suppressed by the blocking of Ca^2+^ channels, indicating that calcium ions play a pivotal role in the development of biofilms. Additionally, Ca^2+^ was involved in forming the cell membrane of *Candida albicans* [41,42,43,44], which corroborated the findings of Momenijavid et al. [40]. In addition, Zhao et al. [45] observed that the Rch1 protein in *Saccharomyces cerevisiae* responded to elevated extracellular Ca^2+^ concentrations and was positively regulated by the calcium/calcineurin signaling pathway. This indicated that the Rch1 protein might influence the permeability of the cytoplasmic membrane by modulating the concentration of calcium ions within the cytoplasm.

Ca^2+^ plays a pivotal role in many processes, such as protection of cells, facilitation of cell division, and reproductive differentiation in microbial cells [46,47,48]. Studies have indicated that the differentiation of the strains is stimulated by the action of Nigericin sodium salt under the regulation of Ca^2+^ [49]. Tisi et al. [50] demonstrated that Ca^2+^ regulated the formation and function of aerial hyphae by activating specific signaling pathways, such as the high-affinity calcium system (HACS) and transient receptor potential calcium (TRPC) channels. These, in turn, affected the growth and pathogenicity of fungi. The genes and related enzymes regulating the absorption and transport of Ca^2+^ within the cells have also been linked to the formation of hyphae and sclerotia [51,52]. Recent research has revealed that Ca^2+^ can alter the cell cycle. The addition of Ca^2+^ was observed to result in a decrease in the expression of cyclins A and B, while an increase in cyclin D expression was noted, which ultimately affected cell division [53].

### 2.2. The Impact of Ca^2+^ Addition on Enrichment Rate in Microorganisms

While the microorganisms are highly effective at accumulating calcium, Ca enrichment capacity varies significantly depending on the species, culturing conditions, Ca sources, and dosages (Table 1). It is notable that the source and dosage of calcium can highly impact calcium enrichment in microorganisms (Table 1). In the research conducted by Wang et al. [54], the enrichment rate of *Wolfiporia cocos* reached only 0.89 mg/g when CaCl_2_ was utilized as a calcium source with a Ca^2+^ concentration of 2.0 g/L. In contrast, Xiong et al. [55] observed that the enrichment of *Inonotus obliquus* was 100.6 mg/g when calcium ions were added at a content of 0.20 mg/L. Recently, Shi et al. [4] studied the calcium accumulation ability of seven kinds of lactic acid bacteria. The findings showed that *Lactobacillus plantarum* CY1-1 and *Lactobacillus plantarum* Z7 exhibited an enrichment capacity of 45.41 mg/g and 37.9 mg/g, respectively, at a CaCl_2_ content of 1.2 mg/L. Moreover, the calcium-rich capacity of the complex strains of *Lactobacillus sake* YP4-5 and *Lactobacillus plantarum* Z7 was found to be up to 52.60 mg/g, which was more pronounced than that observed for the individual strain. The difference in calcium accumulation may be attributed to the biological characteristics and metabolic mechanisms of microbial species. It is, therefore, essential to select appropriate microbial species and to optimize the use of calcium sources in order to improve the calcium enrichment rate. Current reports on calcium enrichment in microorganisms predominantly utilize CaCl_2_, CaCO_3_, and Ca(NO_3_)_2_ as Ca sources (Table 1). In addition, inedible Ca sources have been utilized for calcium enrichment in edible fungi, such as agricultural lime, starfish powder, eggshells, and oyster shells, which contain CaCO_3_ as their primary ingredient [19,56,57]. In the investigation of Choi et al. [56], the authors showed that supplementing 2.0% of oyster shell powder to sawdust medium had the potential to enhance calcium content in the fruiting bodies of *Pleurotus eryngii* (3.16 ± 0.16 mg/g), without affecting the duration of the spawning run, or the days to primordial production. Nevertheless, the addition of more than 4.0% of oyster shell powder to sawdust medium led to a notable reduction in mycelial growth.

### 2.3. The Impact of Ca^2+^ Accumulation on the Metabolites and Secondary Metabolite Production

The exogenous addition of Ca^2+^ has a marked effect on the metabolism of the microbial cells. It has been demonstrated that Ca^2+^ plays a pivotal role in regulating microbial metabolic processes, particularly in enhancing the production of specific metabolites [24,60,61,62]. He et al. [5] found a significant correlation between the production of tropolic acid by sulfur-producing bacteria and the concentration of calcium ions. In the absence of Ca^2+^, the capacity for tropolic acid production was at its maximum. In contrast, when the concentration of Ca^2+^ was higher than 500 mg/L, the yield of tropolic acid declined significantly. However, in the investigation of Wu et al. [38], the authors indicated that the addition of an appropriate amount of Ca^2+^ could effectively increase the synthesis of L-lactic acid by the mycelium of *Rhizopus oryzae*. Similarly, Yue [63] observed that culturing *Ganoderma lucidum* with the appropriate Ca^2+^ content could enhance the accumulation of monomeric ganoderic acids. Furthermore, in the work of Foster et al. [60], the researchers demonstrated the supplementation of CaSO_4_ (gypsum) in the cultivation of *Psilocybe cubensis* led to a notable increase in the yield of the secondary metabolite psilocybin (0.95%). The exogenous addition of Ca^2+^ has also been shown to influence the synthesis of polysaccharides and proteins [64,65]. Adil et al. [64] reported that the presence of Ca^2+^ resulted in an increase in the activity of enzymes involved in polysaccharide synthesis, such as phosphoglucose isomerase, phosphoglucose mutase, and UDP glucose pyrophosphorylase. The mycelial biomass, intracellular polysaccharide, exopolysaccharide, and total polysaccharide content of *Lentinus edodes* exhibited a notable alteration. In microbial cells, Ca^2+^ can bind to relevant proteins to form calmodulin (CaM). The existing investigations have demonstrated that CaM signaling may play a pivotal role in cercosporin biosynthesis, regulate aflatoxin production at the transcriptional level, and regulate the synthesis of glucoheptonate in *Bipolaris maydis* [66,67,68]. Therefore, further studies on CaM-related metabolic activities is beneficial for exploring the pathways through which Ca^2+^ affects microbial metabolism and for the optimal utilization of microorganisms to produce metabolites.

Ca^2+^ regulates a range of physiological functions and metabolic processes within the cells by activating specific calcium-sensing proteins and transcription factors, influencing gene expression, and modulating enzyme activity [69]. Zhang [70] investigated the effects of exogenous Ca^2+^ addition on *Bacillus natto* spore growth through real-time quantitative PCR analysis. The findings revealed that Ca^2+^ significantly influenced glutamate dehydrogenase activity, cell growth, yield, and substrate consumption during *Bacillus* natto fermentation. Specifically, Ca^2+^ enhanced the activity of glutamate dehydrogenase through regulatory mechanisms in vivo. This process might entail the regulation of glutamate dehydrogenase gene transcription levels by Ca^2+^, thereby increasing enzyme expression. Similarly, Ren [61] also reported that Ca^2+^ could promote the transcription of glutamate dehydrogenase-related genes at the α-ketoglutarate node in *Bacillus* natto spores. Nevertheless, the precise molecular mechanism by which Ca^2+^ influences gene expression in microorganisms remains elusive.

### 2.4. The Impact of Calcium Accumulation on the Antioxidant System

The intracellular antioxidant enzyme system in microbial cells is a vital component of the microbial growth process [71]. Upon entering into the cells, Ca^2+^ absorbed by the cells can, through binding to relevant proteins, influence the metabolic activities of the cells. Singh et al. [72] reported that within plant leaves, Ca^2+^ could facilitate the expression of peroxidase (POD), superoxide dismutase (SOD), glutathione S-transferases (GST), and catalase (CAT), thereby enhancing the activity of antioxidant enzymes. The application of Ca^2+^ resulted in a notable enhancement in the activity of CAT while concurrently exhibiting a pronounced inhibitory effect on postharvest POD, polyphenol oxidase, and ascorbate POD. Additionally, the addition of Ca^2+^ may significantly impact the activity of the relevant antioxidant enzymes in microbial cells. Yin et al. [73] reported that the simultaneous influence of exogenous Ca^2+^ and ectomycorrhizal fungi could enhance the activity of antioxidant enzymes, including SOD, in Chinese sea buckthorn seedlings when compared to the control group. This indicated that Ca^2+^ exerted a stimulatory effect on the antioxidant enzyme system in this fungus. Recently, Sun [48] observed that the addition of calcium ions into a yeast culture exposed to high-sugar conditions resulted in the enhanced expression of antioxidant genes, including Mn-SOD, CAT, γ-glutamylcysteinse synthase, glutathione POD, and glutathione transferase. Furthermore, during the 24- and 36-h growth phases, the yeast cells treated with high sugar and calcium ions exhibited a reduction in glycerol and trehalose synthesis, accompanied by an improvement of the intracellular reactive oxygen species (ROS) concentration. However, the yeast demonstrated an enhanced antioxidant capacity through an improvement of SOD and CAT activities, as well as an elevation in glutathione content. These findings suggested that Ca^2+^ played a pivotal role in modulating the antioxidant enzyme activity within microbial cells. Furthermore, Ca^2+^ affected the ability of microorganisms to respond and adapt to adverse environments by influencing the composition and activity of the antioxidant enzyme system [74]. However, in the study of Jin [75], the author investigated the regulation of CaSO_4_ on the growth of *Lentinula edodes*. The addition of 3.0% CaSO_4_ significantly promoted the growth of *Lentinula edodes*, but the expression of manganese POD did not change significantly. The non-enzymatic antioxidant compound is a small-molecule substance secreted by microbial cells that scavenges ROS or other free radicals. At present, there is limited research examining the influence of Ca^2+^ on the non-enzymatic antioxidant activity within microbial cells. However, Zhou et al. [76] observed that Ca^2+^ influenced the antioxidant activity of quercetin in the cells when culturing cells with externally added Ca^2+^. As the concentration of Ca^2+^ increased, the activity initially increased, then decreased, and finally recovered. The findings offered a potential avenue for further research into the non-enzymatic antioxidant substances present within microbial cells.

## 3. Mechanisms of Calcium Transport and Enrichment in Microbial Cells

Ca^2+^ serves as vital signaling molecules within microorganisms, with variations in their concentration capable of triggering multiple intracellular response pathways and participating in vital activities such as cell proliferation, division, growth, and differentiation [48,77]. Currently, a considerable number of investigations have been conducted on Ca^2+^ transport pathways in microbial cells, finding regulatory mechanisms for intracellular Ca^2+^ levels and various Ca^2+^ channel transporters. Nevertheless, in contrast to the extensive research on Ca^2+^ transport mechanisms, there has been limited investigations into Ca^2+^ accumulation mechanisms.

### 3.1. Calcium Transport and Enrichment in Eukaryotic Microorganisms

#### 3.1.1. Adsorption of Ca^2+^ by Extracellular Matrix

The efficiency of the cells utilizing Ca^2+^ is significantly related to the adsorption capacity of Ca^2+^ on the cell membrane surface. Free Ca^2+^ binds to the receptors on the cell surface, subsequently entering into the cell through the pathways, such as Ca^2+^ channel proteins. However, when the receptors are bound solely through free diffusion, the rate of change in the concentration of Ca^2+^ within the fungal cell is relatively slow, as is the response rate. Consequently, the cell surface frequently comprises extracellular polymeric substances (EPS), which facilitate the adsorption of Ca^2+^ and accelerate the change in Ca^2+^ signaling within the cells. Zhao and Jie [25] reported that the EPS produced by edible fungi was primarily composed of polysaccharides that adhere to or surround the mycelial surface of edible fungi. Furthermore, it plays a pivotal role in maintaining the biological morphology of edible fungus cells, the secretion of extracellular enzymes, and the defense mechanism against external interference, including heavy metal ions. However, there are currently limited investigations on the adsorption of Ca^2+^ by EPS, with the majority of researchers focusing on the absorption of heavy metal ions by EPS [78,79]. Many investigations have indicated that the adsorption efficiency of EPS can be influenced by various factors, including cultivation temperature, pH, metal concentration, and the type of culture medium employed [80,81,82]. These findings provided preliminary insight into the molecular-level mechanism by which EPS absorbed metal ions and offered a basis for further research on the pathway by the microbial cells absorbing Ca^2+^.

#### 3.1.2. The Pathways Involved in Calcium Transport and Enrichment Within Eukaryotic Microorganisms

A gradient of approximately 20,000-fold was observed between the intracellular calcium level of around 100 nM (nanomoles per liter) and the extracellular calcium level in the millimole per liter (mM) range [83]. This indicated that the concentration of Ca^2+^ within the cytoplasm was typically maintained at a low level to sustain this concentration gradient, thereby enabling the cell to utilize Ca^2+^ for efficient signaling. The changes in Ca^2+^ concentration result in the transduction of intracellular signals, thereby facilitating a range of biological processes. At present, a number of mechanisms for Ca^2+^ absorption and transport, along with the associated proteins and genes, have been identified in eukaryotic cells.

##### Ca^2+^ Transport System

The maintenance and regulation of intracellular Ca^2+^ levels in eukaryotic cells is primarily achieved through two mechanisms. The first involves the absorption of Ca^2+^ from the extracellular environment via channel proteins on the plasma membrane. The second mechanism entails the mobilization of stored Ca^2+^ within the cells [84]. Among these, it has been observed that in fungal cells, the system for absorbing Ca^2+^ from the extracellular environment via the plasma membrane primarily consists of two major classes: HACS and the low-affinity calcium ion absorption system (LACS). Additionally, both are involved in the regulation of key physiological processes such as growth, development, and pathogenicity in fungi [85]. Fungi typically initiate the activation of their Ca^2+^ absorption and transport systems in response to external environmental and intracellular factors. For example, a study conducted by Stefan et al. [86] revealed that extracellular K^+^ and transmembrane proteins Kch1 and Kch2 were indispensable elements for HACS activation during the cultivation of *Saccharomyces cerevisiae*. In response to mating pheromones, the cell over-expressed Kch1 and Kch2, which were localized to the plasma membrane and activated the HACS in a manner dependent on extracellular K^+^ rather than pheromones. This facilitated Ca^2+^ absorption, enabling participation in intracellular physiological activities. In contrast to the HACS, which is activated when Ca^2+^ availability is low, LACS plays a role in acquiring Ca²⁺ from the extracellular environment during conditions of high extracellular calcium concentrations [87]. In regard to calcium accumulation in edible mushrooms, it has been observed that as Ca^2+^ content increases, Ca^2+^-ATPase, a calcium transport system located on the cell membrane, is activated in a corresponding manner. He et al. [3] identified the presence of Ca^2+^-ATPase on the cell membrane of the mycelium of edible mushrooms, which could regulate the efficiency of Ca^2+^ absorption by the mycelium. Upon increasing the Ca^2+^ addition to a value exceeding the threshold, the activity of Ca^2+^-ATPase was found to be inhibited, resulting in a decrease in Ca^2+^ absorption and transport. This ultimately led to a reduction in calcium accumulation in the mycelia [3]. Furthermore, Bu et al. [59] reported that the typical enrichment of calcium was accomplished with the assistance of Ca^2+^-ATPase, as calcium ions were unable to pass freely through the hydrophobic membrane of edible mushrooms. For the majority of non-excitable cells, the primary mechanism for Ca^2+^ absorption is store-operated calcium entry [88,89]. This process is initiated by a reduction in intracellular calcium concentrations, which activates the endoplasmic reticulum (ER)-resident calcium sensor protein STIM. This, in turn, gates and opens the Orai calcium channels located on the plasma membrane, thereby facilitating the influx of external Ca^2+^ into the cells [90].

The ER functions serve as a Ca^2+^ depository in eukaryotic cells. Recent findings have indicated that mitochondria can couple with the ER, modulating the absorption and release of Ca^2+^ through the activation or inhibition of specific channels. This process maintains the concentration of Ca^2+^ within the cytoplasm at a low level of 100 nmol/L [91] and is involved in critical Ca^2+^ transport pathways within the cytoplasm, serving as a crucial mechanism for the mobilization of intracellular calcium reserves [92]. The ER releases Ca^2+^ into the cytoplasm via the 1,4,5-triphosphate inositol and ryanodine receptors, thereby contributing to vital biological processes [92]. In the event of low Ca^2+^ levels within the ER lumen, the Stromal interaction molecule one protein receives the signals, which are then conveyed to the ER to activate Orai proteins [93]. Subsequently, Orai proteins facilitate the formation of calcium release–activated channels through their passage through the plasma membrane, thereby enabling the uptake of extracellular Ca^2+^ into the cytoplasm [94]. The ER Ca^2+^ pump then transports Ca^2+^ into the ER against a concentration gradient by consuming ATP, thereby maintaining a stable concentration of Ca^2+^ within it [95]. In contrast, it is generally accepted that mitochondria form channel pores only upon oligomerization of mitochondrial calcium uniporter (MCU), which then regulates the opening and closing of these pores through other structural components. However, recent research by Yamamoto [96] using yeast recombinant technology has demonstrated that the mitochondrial calcium single transport protein interacted with the essential MCU regulatory subunit, resulting in a stable open state of the calcium channel and an enhanced mitochondrial calcium uptake capacity.

##### Calcium Channel Proteins and Genes

**Figure 1:**
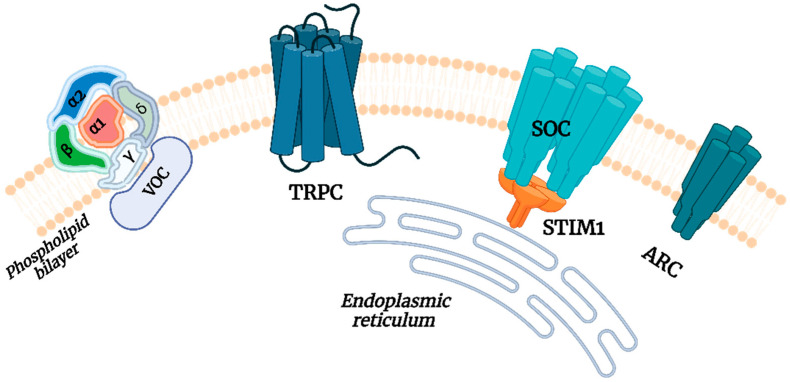
The typical Ca^2+^ channels. Voltage-operated calcium (VOC) channels, transient receptor potential calcium (TRPC) channels, store-operated calcium (SOC) channels, and acid-regulated calcium (ARC) channels.

Ca^2+^ channels are of great importance in the cellular uptake of Ca^2+^ and the maintenance of Ca^2+^ concentration. The current research has indicated that the principal types of Ca^2+^ channels in eukaryotic microorganisms are receptor-operated calcium (ROC) channels, VOC channels, stretch-activated calcium channels, SOC channels, and TRPC channels, among others [84,97]. A representative channel structure is illustrated in Figure 1.

**Figure 2:**
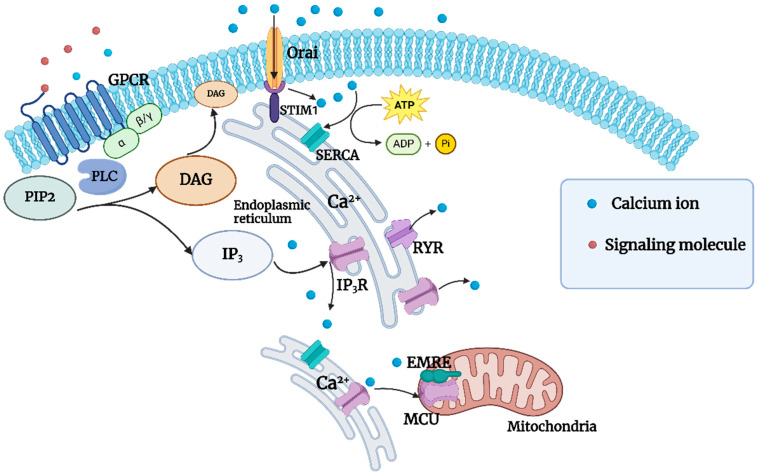
The common Ca^2+^ transport pathways in eukaryotic cells.

Ligand-gated channels are commonly found in neural cells, while voltage-gated channels represent a common method of Ca^2+^ absorption in the majority of eukaryotic cells [98]. The VOCs reported here can be categorized into six types based on their transmitted current characteristics: N, Q, P, T, R, and L. These voltage-gated Ca^2+^ channel proteins primarily consist of four or five subunits (α1, α2, β, γ, δ). Among their structural components, the α1 subunit plays a pivotal role in forming the pore, which is essential for voltage sensors, gating circuits, and intracellular regulatory target regions [99]. Fischer et al. [100] found a crucial Ca^2+^ channel complex in the plasma membrane of yeast cells. This complex, composed of Cch1, which was homologous to the α1 subunit of mammalian voltage-gated calcium channels, represented a significant advancement in our understanding of cellular calcium signaling. Another component, the Mid1 protein, has been identified as belonging to a stretch-activated Ca^2+^ channel [101]. The complex, composed of Cch1 and Mid1, played a significant role in the Ca^2+^ absorption pathway of fungi, which was in accordance with the findings of Revel et al. [102]. However, when amiodarone was added exogenously into the culture *Candida albicans* to stress the Cch1/Mid1 complex and inhibit its Ca^2+^ uptake capacity, it was found that the uptake of Ca^2+^ was not completely eliminated. This suggested the existence of other absorption pathways, which provided insights into the Ca^2+^ absorption pathways in yeast cells. In a comprehensive investigation, Feske et al. [103] elucidated the intricate interactions of the STIM/Orai complex, culminating in the formation of a calcium transport channel for SOC storage operations (Figure 2). The intracellular calcium signaling process, whereby phosphatidylinositol bisphosphate (PIP_2_) is hydrolyzed by phospholipase C (PLC) to produce inositol trisphosphate (IP_3_) and diacylglycerol (DAG). Subsequently, IP_3_ triggers the release of stored calcium ions from the ER via the IP_3_ receptor, resulting in an elevation of the cytosolic Ca^2+^ concentration (Figure 2). This rise in intracellular calcium concentration activates downstream signaling molecules, such as protein kinase C (PKC), which are involved in cellular signal transduction and regulation. Additionally, the activation of receptors on the cell membrane leads to the participation of G-protein-coupled phospholipase C in this process, thereby facilitating the generation of DAG and the release of Ca^2+^.

In recent research, a novel vector has been identified that exhibits sensitivity to nifedipine and gadolinium and is activated by glucose [104]. Furthermore, this vector is involved in activating the plasma membrane H^+^-ATPase in response to glucose. Through culturing the strains with single-gene deletions of YVC1 or double-gene deletions of YVC1 and PMC1, the researchers observed that the glucose-induced calcium signaling was almost completely eliminated, and the H^+^-ATPase also became inactivated. This indicated that the YVC1 Ca^2+^ channel played a pivotal role in the signal transduction pathway initiated by glucose addition. Moreover, it has been demonstrated that culturing the cells with glucose could influence the uptake of Ca^2+^ uptake and the Ca^2+^ channel YVC1 activity, thereby triggering calcium signaling. This offered a novel research avenue for research into calcium transport pathways in eukaryotic cells. The expression and regulation of various calcium channel proteins and calcium signaling are governed by intracellular calcium signaling genes [105,106,107,108], yet the regulatory mechanisms remain poorly understood.

##### Calcium-Binding Proteins and Intracellular Storage Modes

**Figure 3:**
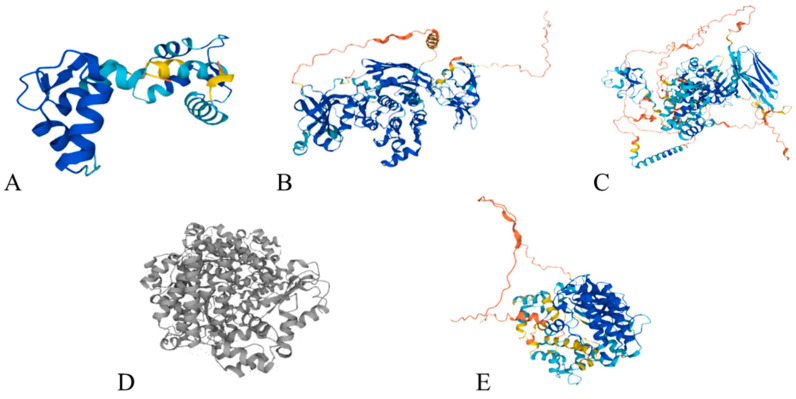
The common calcium-binding proteins in eukaryotic organisms. (**A**) calmodulin (CaM) from *Pongo abelii*; (**B**) protein kinase C (PKC) from *Caenorhabditis elegans*; (**C**) CaM from *Caenorhabditis elegans*; (**D**) calcineurin B-like protein (CBL) from *Homo sapiens*; (**E**) calcium-dependent protein kinase (CDPK) from *Solanum tuberosum*.

In eukaryotic cells, the ER typically absorbs and stores a substantial quantity of free Ca^2+^ via calcium release-activated calcium (CRAC) channels, thereby maintaining a low concentration of calcium ions within the cytoplasm [109]. The release of Ca^2+^ occurs when the signals are transmitted within the cells, and it functions in conjunction with calcium-binding proteins to regulate cellular activities. A number of calcium-binding proteins have been identified, including calcineurin B-like protein (CBL), protein kinase C (PKC), calcium-dependent protein kinase (CDPK), and CaM (Figure 3). Each protein participates in distinct reaction processes, with CaM being the most extensively studied [110,111].

CaM contains four “EF-hand”-type structures and is capable of binding Ca^2+^. The interaction between CaM and target proteins is dependent upon the number of Ca^2+^ bound to the proteins, specifically one Ca^2+^ for the death-associated protein kinase peptide, two calcium ions for the probe, and four calcium ions for the EGFR peptide [112]. The investigation has indicated that CaM can bind to cation-binding protein 1, thereby influencing calcium signaling transduction [113]. Moreover, calcium-binding proteins CBLs, which are found in eukaryotic cells, contain four conserved EF-hand structures, are capable of binding Ca^2+^, and induce cellular localization [114]. CBLs interact with CBL-interacting protein kinases, modulating kinase activity in response to extracellular signals, is consistent with the reports by Tang et al. [115]. Additionally, Ca^2+^ taken up into eukaryotic cells can also be absorbed by mitochondria, where they accumulate in the mitochondrial matrix to form precipitates, which are released during signal transmission [116].

### 3.2. Calcium Transport and Enrichment in Prokaryotes

Prokaryotic microorganisms, like lactic acid bacteria, distinguished by their rapid growth and low cultivation costs, have been extensively employed in industrial production. However, in comparison to eukaryotic microorganisms, prokaryotes possess a simpler structure and lack ER, mitochondria, and other calcium-storing organelles within the cytoplasm, which results in reduced efficiency in the absorption and storage of Ca^2+^. The current research has identified a number of Ca^2+^ channel proteins and calcium-binding proteins in prokaryotes, which provides a basis for further investigation into the mechanisms by which prokaryotes enrich calcium ions. This also has the potential to enhance the capacity of prokaryotes to concentrate Ca^2+^ at the molecular level, thereby reducing industrial production costs.

#### 3.2.1. Ca^2+^ Channel Protein Within Prokaryotic Cells

**Figure 4:**
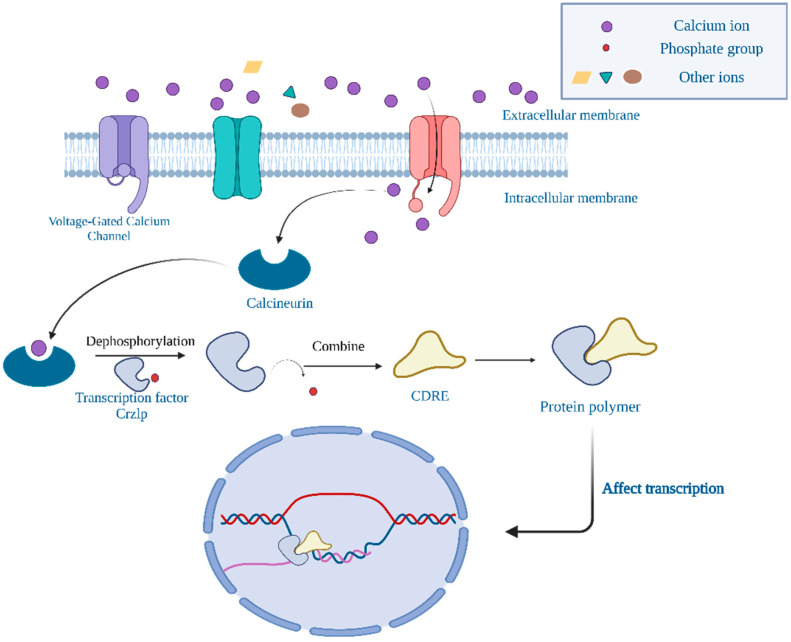
The intracellular Ca^2+^ transport channel in *Candida albicans*.

Ca^2+^ plays a vital role as signaling molecules in living organisms. Recent research has indicated that the microbial cells maintain a relatively low Ca^2+^ content in the cytosol through the regulation and transport of Ca^2+^. In prokaryotic microorganisms, Ca^2+^ is primarily transported through structural proteins, including ion channel proteins and ion pumps. Brand et al. [117] identified Ca^2+^ channel voltage-gated membrane proteins Cchlp and Midlp on the cell membrane of *Candida albicans* and investigated their involvement in calcium influx (Figure 4). In *Candida albicans*, calcium ions can enter cells through voltage-gated calcium channels and mechanically sensitive calcium channels. The activation of these channels may be associated with the polarization of the cell membrane and extracellular signals, including electric fields and surface topology. The influx of calcium ions results in local alterations in intracellular calcium concentrations, which may affect intracellular signaling pathways, including the activation of calcineurin and the transcription factor CaCrz1p (Figure 4), which regulate gene expression and cellular behavior. Furthermore, calcium ions may be directly involved in cell polarity growth and directional responses, such as electric field tropism and tactile tropism, which are crucial for the fungal tissue penetration and infection processes [117]. The possible role of phosphate groups in Ca^2+^ transport and cell signaling is also illustrated in Figure 4.

In early research, it was noted that sodium ion channel proteins were present in all living organisms. Furthermore, no calcium channel proteins that resemble eukaryotic cells were identified on prokaryotic membranes. However, a comparison of voltage-dependent Ca^2+^ channels in eukaryotic cells with sodium ion channels in prokaryotes revealed the similarities in structure and evolutionary history [118]. This observation gave rise to the hypothesis that the absorption of calcium ions by prokaryotes could be selectively facilitated through the modification of the BACNAV gene [119]. Additionally, a non-protein compound, poly-β-hydroxybutyrate-polyphosphate, was identified in *Escherichia coli* by Pavlov et al. [120]. This compound had the capacity to form a non-protein Ca^2+^ channel, which facilitated the transport of Ca^2+^ into the cytoplasm. This represented an unconventional method of Ca^2+^ absorption observed in prokaryote cells. However, a recent comparative analysis of genes across multiple organisms has identified a voltage-dependent Ca^2+^ channel CavMr in *Meiothermus ruber* [119]. This represented the inaugural discovery of a Ca^2+^ channel in prokaryote cells. Subsequent experiments conducted by this research group demonstrated that the glycine residue within the CavMr selectivity filter was the critical determinant for Ca^2+^ selectivity, providing substantial evidence for future investigation into Ca^2+^ channels in prokaryotes [121]. Meanwhile, the P-type ATPase pump SERCA and ATPases involved in Ca^2+^ transport have been confirmed in prokaryote cells [121,122].

In brief, the current understanding of the transport and regulation of Ca^2+^ in eukaryotic cells is relatively comprehensive. However, prokaryotic cells lack ER as a calcium storage pool and exhibit membrane structures that differ from those of eukaryotes. While there have been a few research reports on related Ca^2+^ absorption and transport proteins, the specific mechanisms remain unclear.

#### 3.2.2. Ca^2+^ Adsorption and Accumulation of Through the Surface Group of Prokaryotics

In comparison to eukaryotic microorganisms, prokaryotic microorganisms possess a simpler morphological structure and primarily produce EPS that are involved in physiological activities. Nevertheless, the research has demonstrated that the constituents of EPS produced by prokaryotes are intricate and serve a multitude of functions, predominantly associated with the adhesion of microbial strains, the growth of biofilms, and the adsorption of metal ions [123,124]. The absorption of calcium ions by microbial strains has been carried out. Shi et al. [4] reported that a significant quantity of calcium ions was observed to accumulate on the surface of the bacteria after the enrichment of calcium ions with lactic acid bacteria, resulting in a great difference in cell morphology from normal strains. The EPS also possesses a high content of anions and the ability to chelate calcium ions, which is crucial for the absorption of calcium ions by microbial strains [125]. In the work of Braissant et al. [126], EPS extracted from sulfate-reducing bacteria were placed in a solution containing Ca^2+^ at pH 9. The results demonstrated that the amount of adsorbed Ca^2+^ per gram of EPS could reach 0.12–0.15 g. Concurrently, the authors observed that the adsorption of calcium ions by bacterial strains’ EPS could inhibit the transmission of mamp-induced signals in their host plants, thereby providing a novel strategy for establishing resistance against pathogenic bacteria. Nevertheless, the research indicated that calcium ions were still adsorbed outside the cells in the absence of EPS, suggesting that EPS might be one of several extracellular structures with relevant adsorption capabilities. The study is required to gain a deeper understanding of the adsorption of calcium ions by bacterial strains in the future [124].

#### 3.2.3. Ca^2+^-Binding Compounds Within the Prokaryotic Cells

In eukaryotic cells, calcium ions are often present in a free state within the ER lumen. Upon the reception of signaling molecules, exogenous calcium ions are absorbed and released from the ER lumen, thereby increasing the concentration of calcium ions in the cytosol. The calcium ions that enter the cytoplasm participate in a variety of metabolic activities within the cells, primarily by binding to CaM, EF-hand, and other proteins to regulate processes such as cell proliferation and signal transmission [127,128]. In recent years, investigations have also been conducted on the storage and regulation mechanisms of calcium ions in prokaryotic cells. Yang [129] identified diverse CaM proteins in *Streptomyces*. Similarly, Kayastha et al. [130] reported that the Ca^2+^-binding protein EfhP, due to its structure, contained two typical EF-hand motifs, which were capable of effectively binding to calcium ions and regulating the virulence factors and infectivity of the bacteria. Proteins that exhibit cellular activity possess distinctive secondary and tertiary structures. Hoyer et al. [131] observed that the MIIA domain of homologous proteins in *Vibrio corallii* was disordered and non-active. However, upon binding to calcium ions, this domain underwent a structure change, resulting in the acquisition of specific functional activity. Martínez-Gil et al. [132] also demonstrated that calcium ions could bind to protein domains, inducing structural alterations in proteins and facilitating related biological processes. In prokaryotic cells, the absence of ER precludes the storage of free calcium ions. Following the absorption of calcium ions by prokaryotic cells, a minor quantity of calcium ions would bind to relevant proteins, thereby expressing activity and regulating metabolism. A greater quantity of calcium ions would combine with specific proteins, polysaccharides, nucleic acids, and other biopolymer active groups to form biological inclusions or chelates, thereby converting them into organic calcium, which is more beneficial for biological utilization [19,133]. Nevertheless, there are few studies investigating the calcium enrichment mechanisms of prokaryotic cells that are capable of effectively converting inorganic calcium into organic calcium.

## 4. Developments of Microbial Calcium-Fortified Products

Many researchers have employed the utilization of filamentous fungi, lactic acid bacteria, yeast, and other microorganisms to accumulate numbers of trace elements. The findings have indicated that the microorganisms exert a pronounced influence on the accumulation and transformation of calcium, zinc, selenium, chromium, and other elements [19,134,135,136,137]. The accumulation of trace elements can enhance their stability and facilitate their transformation into organic forms that are more readily absorbed and utilized by the human body [138]. Presently, the main microorganisms employed for the development of calcium-rich products are edible fungi, yeast, and bacteria (Table 2).

### 4.1. Calcium Enrichment by Edible Fungi

The cultivation of edible fungi has a long history in China. Studies have indicated that edible fungi display a notable bioaccumulation capacity for calcium, germanium, zinc, iron, selenium, and other essential elements. The trace elements within edible fungi enhance their chemical stability and facilitate their conversion into an organic form, which is characterized by safety, high absorption rates, and the absence of toxic or adverse effects [19,138]. The calcium enrichment facilitated by edible fungi yields organic calcium or amino acid calcium variants that exhibit enhanced bioavailability to the human body. With appropriate processing, the enriched calcium products have the potential to serve as raw materials for novel calcium supplements, with significant developmental prospects. The calcium content within mycelium through the supplementation of calcium in the growth medium provides a foundation for the advancement of functional calcium supplements [19]. *Flammulina velutipes* is a readily available and rapidly growing species. In the study of Fang et al. [22], the combination of calcium and edible fungi was achieved by enriching calcium using *Flammulina velutipes* mycelium. The authors employed *Pleurotus ostreatus* and *Flammulina velutipes* to produce calcium-enriched mycelium through liquid fermentation. The mycelium was subjected to hydrolysis with protease, and the resulting hydrolysate was added to sour soymilk to create a calcium-rich food product. The production of calcium-rich sour soymilk using *Flammulina velutipes* was characterized by a short production cycle, low cost, and high added value. However, the authors found that the hydrolysis of mycelium by hydrolase and its subsequent addition to soy milk might result in the loss of Ca^2+^ and a reduction in the calcium-rich rate. Following the work by Kang et al. [139], the sponge carrier, composed of konjac glucomannan, was inoculated with *Pleurotus eryngii* and combined with calcium chloride as the calcium source. The resulting konjac edible fungus calcium-rich functional beverage was then prepared. This method enriched calcium through the use of an edible carrier culture and a low-temperature drying method, which reduced the loss of Ca^2+^ and significantly improved the calcium enrichment rate of the strains. It was anticipated that this method would evolve into a novel fermentation technology in industry, thereby enhancing the calcium enrichment capacity of the strains. The findings of this research not only showed the potential applications of calcium-enriched edible fungi in the food industry but also highlighted the adaptability and growth characteristics exhibited by diverse fungal mycelia in response to varied calcium sources.

Recent studies have demonstrated the substantial influence of Ca^2+^ on the growth and metabolic processes of fungal strains. In a fermentation broth containing 200 μg/mL of Ca^2+^, Xiong et al. [55] observed that *Ganoderma lucidum* exhibited peak extracellular polysaccharide content and Ca^2+^ levels. Concurrently, Wang et al. [54] found that when the mass concentration of CaCl_2_ was less than or equal to 2.0 g/L, the growth of the mycelium was not impeded, whereas, when the mass content exceeded 2.0 g/L, the growth rate of the mycelium slowed down and the density decreased. This demonstrated that the Ca^2+^ content of the fermentation broth influenced the growth of the strain, and a poor growth state of the strain would consequently decrease its value. It is, therefore, imperative for the researchers to determine the optimal Ca^2+^ concentrations that facilitate the growth of various edible fungi strains with high calcium enrichment rates. The purpose of this endeavor is to refine production technologies for calcium-rich edible fungi by identifying conditions that foster robust strain growth, maximize their nutritional value, and enhance their commercial viability.

### 4.2. Calcium Enrichment by Yeast

As a microorganism with high nutritional value, yeast contains more than 50% protein and abundant physiologically active substances, including amino acids, vitamins, and ergosterol. Some studies have indicated yeast cells possess the ability to enrich calcium ions, convert inorganic compounds into organic compounds, and enhance bioavailability and safety [141,145]. The utilization of high-calcium edible yeast as a novel and efficacious source of calcium offers the advantage of combining the characteristics of both calcium and yeast, thereby facilitating its adsorption. The findings of Zhang et al. [141] suggested that the incorporation of optimal quantities of calcium salts into the culture medium could markedly elevate the calcium content within the yeast cells without affecting the overall yield. Furthermore, Shi and Yan [135] investigated the application of calcium-rich yeast in bread-making. Their findings indicated that moderate calcium supplementation could significantly elevate the calcium content in bread while also improving the apparent digestibility compared to the case of inorganic salt addition. In recent years, researchers have successfully enriched calcium within yeast cells through the screening of calcium-rich microbial resources and high-calcium-tolerant transformant strains. The approach described was environmentally friendly and safe for the development of novel calcium supplements [21].

### 4.3. Calcium Enrichment by Bacterial Cells

Bacteria have the capacity to absorb various kinds of metal ions, attaching them to their surfaces or transferring them into their bodies for accumulation. This property enables bacterial cells to enrich and utilize calcium ions [1,4,146]. It has been demonstrated that there are differences in the adaptability to calcium sources among various bacterial species. For example, lactic acid bacteria demonstrate a markedly elevated calcium enrichment when employing calcium chloride as a calcium source, in comparison to calcium carbonate and calcium phosphate. This variability in adaptability offers a framework for the selection of culture media and raw materials that enhance bacterial calcium enrichment [147]. The combination of probiotic fermented milk with enhanced calcium content may serve as an effective calcium carrier. Szajnar et al. [148] produced a calcium-rich probiotic fermented milk product with high calcium content by adding calcium salts to pasteurized milk and inoculating it with Bifidobacteria. In comparison to the control group, the milk exhibited the highest acidification when calcium chloride and lactate were added, while the addition of calcium glycinate resulted in a notable increase in pH value and alkalinity, leading to the highest bacterial count. As demonstrated by Ju [142], high-calcium milk formulated with calcium citrate and calcium carbonate as calcium sources exhibited superior heat stability compared to milk with calcium lactate as the calcium source. Furthermore, high-calcium milk formulated with calcium citrate as the calcium source demonstrated enhanced heat stability compared to milk with calcium carbonate as the calcium source. Additionally, the calcium in calcium citrate had a distinctive absorption mechanism, with high bioavailability, minimal dependence on gastric acid secretion, and a negligible impact on the intake of other nutrients.

The incorporation of growth-promoting factors has been demonstrated to enhance the calcium conversion rate in fermented cow bone powder through the use of complex probiotics. This finding presented a novel approach for improving production techniques for bacterial calcium enrichment [149]. At present, the research is predominantly concentrated on the fermentation of bacterial microorganisms and the fermentation of bone powder for the purpose of calcium enrichment [143,150,151]. The use of the processing by-product bones for calcium-rich fermentation offers several advantages, including low cost and promotion of sustainable development. Chen [23] employed the use of *Leuconostoc mesenteroides* for the fermentation of grass carp bone fermentation broth, with the objective of obtaining protein and calcium resources. The findings indicated that, in comparison to the enzymatic broth derived from fish bones and calcium lactate, the fish bone fermentation broth exhibited a more significant effect in providing calcium supplements. This suggested that a composite of organic acid calcium, peptide calcium, free organic acids, and amino acids might possess a greater capacity to promote calcium absorption than a single peptide calcium or organic acid calcium. Wang [143] employed the use of *Lactobacillus reuteri* for the fermentation of pacific cod bone, resulting in the production of a lactic acid bacteria calcium supplement. As a novel health food, the calcium supplement product containing lactic acid bacteria offered one way to enhance the comprehensive benefits and economic added value of processing fish bone waste materials. Tang [144] conducted lactic acid fermentation on bone powder, which revealed that the lactic acid bone powder fermentation broth was enriched with significant amounts of free calcium and that the calcium-to-phosphorus ratio in the broth was more suitable for human absorption [152]. In summary, bacterial calcium enrichment encompasses not only the selection of an appropriate medium and raw materials but also the examination of bacterial adaptability to different calcium sources and the regulation of biological activities. The findings of these studies provide substantial evidence for supporting the development of bacterial calcium supplement preparations.

## 5. Conclusions

Calcium is a vital component of the human body, playing a pivotal role in its composition and maintaining overall health. The adequate intake of calcium can serve to prevent a number of diseases, including hypertension, dental caries, osteoporosis, and colon cancer. The growing consumer demand for calcium supplements has driven the expansion of the calcium-based health foods market. Microbial enrichment and transformation techniques have been employed to produce organic calcium-rich products, which has emerged as a cutting-edge area of research in the field of calcium supplementation.

The beneficial microorganisms, such as edible fungi, yeast, and bacteria, have the capacity to accumulate and transform calcium, thereby representing a potential source of calcium-rich foods. Nevertheless, the current studies on calcium-rich foods still encounter many obstacles, such as low calcium enrichment efficiency, slow strain growth, and high production costs. In order to promote better development of calcium-rich products, much work should be carried out in the future. Other than optimization strategies, including fermentation conditions, media conditions, and strain screening, the application of genetic engineering and advanced technologies can also facilitate the enhancement of microbial calcium enrichment capability. These beneficial attempts would establish a theoretical foundation for enhancing and refining calcium enrichment processes. At present, the accumulation mechanisms and physiological impacts of diverse forms of calcium supplements in microbial cells are limited. Changes in calcium ion concentration can trigger a multitude of intracellular reaction pathways, influencing cellular proliferation, division, growth, and differentiation, among other vital processes. A comprehensive understanding of the transport pathways of calcium ions within the microbial cells, encompassing calcium ion channels, pumps, and CaM, has the potential to elucidate the role of calcium in intracellular signaling transmission and regulation networks. Therefore, a deeper understanding of the metabolism and regulatory mechanisms of Ca^2+^ within microbial cells should be carried out to find more efficient strategies for calcium enrichment. Concurrently, an investigation into the correlation between calcium intake and conditions, such as osteoporosis and other ailments linked to calcium deficiency, can facilitate the development of targeted guidance for their prevention and treatment. In the future, by increasing public awareness of the importance of calcium intake, promoting optimal dietary combinations, and educating people on calcium supplementation techniques, it is possible to enhance overall population health.

In conclusion, the enrichment of calcium within the microbial cells has significant implications for human health and nutritional fortification. Future research should prioritize the refinement of the characteristics and production procedures of calcium-rich commodities, as well as an in-depth investigation into the metabolic intricacies and regulatory frameworks governing calcium in microbial cells. This comprehensive approach would facilitate the development of more efficacious calcium supplementation methods and products, which ultimately enhance individual well-being and health outcomes.

## Figures and Tables

**Table 1:** Effect of calcium addition on enrichment rate in microorganisms.

The Strains	Experimental Conditions	Enrichment Rate	Reference
*Wolfiporia cocos*	Ca Source: CaCl_2_; the optimized Ca content for enrichment: 2.0 g/L.	0.89 mg/g	[54]
*Ganoderma lucidum*	Ca Source: CaCl_2_; the optimal Ca content for enrichment: 200 μg/L.	100.6 mg/g	[55]
*Inonotus obliquus*	Ca Source: Ca(NO_3_)_2_; the optimal Ca concentration for enrichment: 1000 mg/L.	21.0 mg/g	[58]
*Pleurotus* *nebrodensis*	Ca Source: CaCl_2_; the optimal Ca concentration for enrichment: 6000 mg/L.	790.6 mg/g	[59]
*Cordyceps sinensis* CCTCC AF99009	Ca Source: Ca(NO_3_)_2_ + CaCO_3_; the optimal Ca concentration for enrichment: 3.0 g/L.	0.78 mg/g	[20]
*Lactobacillus plantarum* CY1-1	Ca Source: CaCl_2_; the optimal Ca concentration for enrichment: 1.2 mg/L.	45.41 mg/g	[4]
*Lactobacillus plantarum* Z7		37.9 mg/g	
*Hypsizygus marmoreus*	Ca Source: CaCl_2_; the optimal Ca concentration for enrichment: 100 mg/L.	22.40 mg/g	[9]
*Lactobacillus fermentum*	Ca Source: CaCl_2_; the optimal Ca concentration for enrichment: 1.2 mg/L.	41.90 mg/g	[1]
*Pediococcus acidilactici*		41.90 mg/g	
*Lactiplantibacillus plantarum*		52.60 mg/g	
*Laetiporus sulphureus (Fr).* Murrill	Ca Source: CaCl_2_; the optimal Ca concentration for enrichment: 100 mg/L.	18.34 mg/g	[5]

**Table 2:** Microbial calcium-rich products.

Categorization	Offerings	Microorganisms	Specificity	Calcium Content	References
Edible Fungi	Calcium-enriched almond mushroom mycelium sour soya bean milk	*Pleurotus eryngii*	Short production cycle, simple equipment, low input cost	47 mg/100 g	[139]
	Calcium-rich spotted mushroom	Mottled mushroomYH01	Good calcium enrichment, absorption, and conversion capacity	/	[140]
	Calcium-rich *Poria cocos* mycelium	*Poria cocos Po*	Rich in calcium while stimulating the production of large amounts of polysaccharides	8911 mg/100 g	[54]
	Calcium-rich *Ganoderma lucidum* hyphae	*Ganoderma lucidum* 730	Good stability; binds to protein, sugar, and fat substances, facilitates absorption and utilization	364.2 mg/100 g	[55]
	Calcium-rich *Ganoderma lucidum*	Red *Ganoderma lucidum*	Organic calcium was *Ganoderma lucidum* polysaccharide calcium	453.82 mg/100 g	[138]
Yeast	Calcium-rich bread	Beer yeast	High apparent digestibility and high bioavailability	10.40 ± 1.10 mg/100 g	[135]
	High-calcium edible dry yeast	beer yeast	Combines calcium with yeast, easy absorption	627 mg/100 g	[141]
Bacterium	High-calcium yogurt	Lactic acid bacteria	High bioavailability, low dependence on gastric acid secretion, low impact on other nutrient intake	160 mg/100 mL	[142]
	Lactobacillus calcium tablets	*Lactobacillus reuteri*	The ratio of calcium and phosphorus was suitable for human needs	50 ± 1.03 mg/g	[143]
	Fermented superfine crushed bone powder	Lactic acid bacteria	Maximum density of enriched calcium in the cell wall	148.5 mg/100 g	[144]
